# SBCOC CONSENSUS STATEMENTS: A SCIENTIFIC COMMITMENT TO GOOD MEDICAL PRACTICE

**DOI:** 10.1590/1413-785220263401e310247

**Published:** 2026-08-21

**Affiliations:** 1 Hospital das Clinicas HCFMUSP, Faculdade de Medicina, Universidade de Sao Paulo, Sao Paulo, SP, Brazil.; 2 Hcor, Sao Paulo, Brazil.; 3 Department of Orthopaedics, Hospital Israelita Albert Einstein, São Paulo, Brazil.; 4 Instituto de Cirurgia do Ombro e Cotovelo (ICOC) / Universidade do Estado do Rio de Janeiro (UERJ), Rio de Janeiro, Brazil.

Increasingly, the quality of care in Shoulder and Elbow Surgery is measured by the ability to bring together three elements that do not always point in the same direction: the best available evidence, the surgeon's experience, and each patient's values. This is how Sackett et al. defined Evidence-Based Medicine — not as the mechanical application of clinical trial results, but as the critical integration of these three domains.^1^ In this context, consensus documents produced by scientific societies play a strategic role: they organize the available knowledge and translate it into recommendations applicable to daily practice, particularly where evidence is scarce, conflicting, or difficult to interpret.

This special issue of Acta Ortopédica Brasileira brings together the Consensus Statements of the Brazilian Society of Shoulder and Elbow Surgery (SBCOC), a project initiated during the term of President Marcelo Campos and concluded under the current administration, led by President Eduardo Malavolta. In all, there are ten consensus statements, spanning the main therapeutic dilemmas of the specialty — from rotator cuff tears (double-row repair for large tears, early versus delayed repair for traumatic tears, augmentation with a bioinductive implant, and the treatment of tendinopathy) to anterior shoulder instability (management of bone loss and the number of anchors), as well as arthroplasty and proximal humeral fractures in the elderly, glenohumeral osteoarthritis, and elbow conditions such as epicondylitis. More than a compilation of opinions, each document arose from a critical and structured analysis of the literature, aimed at synthesizing the best available evidence on questions that are frequent, relevant, and often controversial in daily practice. The continuity of the project across administrations reflects the Society's institutional commitment — through its boards, committees, and members — to high-quality scientific work.

Trustworthy recommendations require methodological transparency, critical appraisal of the quality of evidence, and explicit acknowledgment of uncertainty. For this reason, each consensus was conducted as an independent systematic review, with the certainty of evidence assessed using the GRADE approach, under the methodological guidance of Cochrane Brazil — ensuring rigor, reproducibility, and impartiality in the analyses. This process followed internationally established standards for the development of trustworthy guidelines and consensus documents, such as the criteria of the Institute of Medicine, the AGREE II instrument, and the GRADE approach itself.^2–4^ Starting from structured questions relevant to the Brazilian setting, SBCOC specialists translated the evidence into applicable recommendations — in the office, in the operating room, and across the various care settings in which our members work.

Beyond supporting clinical reasoning, the SBCOC Consensus Statements strengthen the physician–patient relationship. By offering a structured synthesis of the evidence — with its benefits, risks, alternatives, and limitations — they help sustain shared decision-making, which is increasingly central to contemporary medicine. Translating science into understandable information is what allows patients to take part, in an informed way, in the choices about their own treatment.^5^

These documents also play an important role in the recognition and defense of professional practice. In an environment marked by growing interference from health insurers, litigation, and demands for technical justification of diagnostic and therapeutic procedures, positions issued by a scientific society offer qualified support for medical practice. It is worth recognizing, however, that guidelines and consensus documents are not neutral instruments: when misused — as rigid criteria for coverage or liability — they can turn against the very clinical judgment they are meant to support.^6^ For this reason, they should be understood as support for decision-making, not a substitute for it, always in the patient's best interest.

With this special issue, the SBCOC reaffirms its mission to promote education, research, scientific updating, and excellence in care in Shoulder and Elbow Surgery. The Consensus project is an institutional milestone: it is born of collaboration among specialists, spans administrations, relies on independent methodological support, and looks to the future. May these documents serve as a reference for our members, a source of consultation for the orthopedic community, and a lasting stimulus toward critical, ethical, and evidence-based medical practice.

## Data Availability

The contents underlying the research are available in the manuscript.
